# Field emission luminescence of nanodiamonds deposited on the aligned carbon nanotube array

**DOI:** 10.1038/srep09379

**Published:** 2015-03-23

**Authors:** Yu. V. Fedoseeva, L. G. Bulusheva, A. V. Okotrub, M. A. Kanygin, D. V. Gorodetskiy, I. P. Asanov, D. V. Vyalikh, A. P. Puzyr, V. S. Bondar

**Affiliations:** 1 Nikolaev Institute of Inorganic Chemistry SB RAS, Novosibirsk 630090, Russia; 2 Novosibirsk State University, Novosibirsk 630090, Russia; 3 Institute of Solid State Physics, Dresden University of Technology, D-01062 Dresden, Germany; 4 Institute of Biophysics SB RAS, Krasnoyarsk 660036, Russia

## Abstract

Detonation nanodiamonds (NDs) were deposited on the surface of aligned carbon nanotubes (CNTs) by immersing a CNT array in an aqueous suspension of NDs in dimethylsulfoxide (DMSO). The structure and electronic state of the obtained CNT–ND hybrid material were studied using optical and electron microscopy and Infrared, Raman, X-ray photoelectron and near-edge X-ray absorption fine structure spectroscopy. A non-covalent interaction between NDs and CNT and preservation of vertical orientation of CNTs in the hybrid were revealed. We showed that current-voltage characteristics of the CNT–ND cathode are changed depending on the applied field; below ~3 V/µm they are similar to those of the initial CNT array and at the higher field they are close to the ND behavior. Involvement of the NDs in field emission process resulted in blue luminescence of the hybrid surface at an electric field higher than 3.5 V/µm. Photoluminescence measurements showed that the NDs emit blue-green light, while blue luminescence prevails in the CNT–ND hybrid. The quenching of green luminescence was attributed to a partial removal of oxygen-containing groups from the ND surface as the result of the hybrid synthesis.

Nanostructured materials consisting of carbon nanotubes (CNTs) and nanodiamonds (NDs) possess a combination of outstanding functional properties of two constituents, e.g. high electrical and thermal conductivity, mechanical and chemical stability, large surface area. The CNT–ND hybrid materials can find many applications in various technologies, as cold cathodes, biosensors, electrochemical electrodes, components of nanoelectronic devices, additives in protective materials, and adsorbents^1^,^2^,^3^,^4^,^5^,^6^,^7^,^8^,^9^. Plasma enhanced (PE) and hot filament (HF) assisted chemical vapor deposition (CVD) are the most common methods of synthesis of the CNT–ND hybrids. Advantage of the CVD method is the simultaneous growth of CNT and ND structures^9^,^10^,^11^,^12^,^13^. However, it is possible to form hybrid material in a few subsequent steps^1^,^14^,^15^. In the work done by Kinoshita et al the first step was a microwave PECVD growth of an array of CNTs using previously deposited iron nanoparticles on a silicon substrate, and in the next step a diamond-like carbon coating was deposited on the CNTs by a radiofrequency PECVD^1^. Shankar et al obtained the CNT–ND composites by HFCVD growth of NDs on the multiwall CNTs pre-dispersed onto a silicon substrate^14^. However, the outer layers of CNTs were significantly etched by hydrogen plasma and there was only a narrow range of concentration of carbon precursor in hydrogen flow allowing production of the NDs and preservation of initial morphology of the CNTs.

One could expect that a CNT–ND material with the CNTs oriented in predominant direction will have anisotropic properties. Aligned CNTs decorated with ND nanoparticles are especially attractive for optoelectronic devices such as light-emitting systems and photovoltaic cells. High aspect ratio of a CNT causes local electric field enhancement near its tip, which may help electron injection into attached semiconducting nanoparticles. Actually, recently we demonstrated appearance of electroluminescence from CdS nanoparticles grown on the CNT array^16^,^17^,^18^. As of now, there are a couple of papers describing synthesis of the CNT–ND anisotropic materials. Diamond-like carbon coating has been deposited on top of vertically aligned CNT film by PECVD method^1^, and the composite film showed enhanced toughness. However, the synthesis did not allow producing the coating on a CNT body, probably, due to closely packing of CNTs in the film and relatively small nanotube diameters (tens of nanometers). The use of vertically aligned micrometer-sized carbon fibers consisting of nested cones of graphene sheets yielded continuous deposition of ultrananocrystalline diamond film on the fiber surface, and the hybrid structures exhibited greatly improved field emission performance compared with the uncoated fibers^15^. Near-edge X-ray absorption fine structure spectroscopy (NEXAFS) examination revealed no changes in the electronic state of carbon fibers after the PECVD diamond film deposition.

CVD approaches for preparation of the CNT–ND hybrids are not readily scalable. There remains a necessity to develop inexpensive and effective methods of synthesis of CNT–ND materials with predetermined structure and specified properties. One of such methods is based on blending of suspended nanoparticles^6^,^7^,^19^,^20^,^21^. A common procedure is the treatment of a substrate with suspension of CNTs and NDs and subsequent drying of the hybrid until complete evaporation of solvent. Spin coating helps in the creation of thin hybrid films^6^,^7^, while electrodeposition improves dispersion of CNTs in a ND matrix^22^. The advantages of the use of the nanoparticle suspensions are processing simplicity, easy change in ratio of the components, and mild conditions, which do not damage the CNT walls. Morphology and properties of a CNT–ND material obtained using this method generally depend on the dispersion and stability of the suspension, which can be tuned choosing appropriate solvent and modifying the nanoparticle surface. Previously, it was demonstrated that the use of dimethylsulfoxide (DMSO) as a solvent for surface-modified detonation NDs provides high stability of the suspension to sedimentation and aggregation^23^,^24^. Recently, carbon-based nanostructures such as NDs^25^,^26^ and carbon dots^27^,^28^ were considered to be a new class of biocompatible luminescent materials. Blue-green field emission electroluminescence was observed from polycrystalline diamond films^26^,^29^.

In the present paper we report for the first time the synthesis of an anisotropic CNT–ND hybrid material by impregnation of a CNT array with a suspension of NDs. It is shown that treatment of multiwall CNT array by DMSO–water suspension of NDs preserves vertical nanotube alignment toward substrate. Functional composition and electronic structure of the CNT–ND hybrid are examined by Fourier-transform infrared (FTIR) and Raman spectroscopy, NEXAFS and X-ray photoelectron spectroscopy (XPS). Testing of CNT–ND hybrid under applied voltage reveals field emission electroluminescence of ND particles located at the array surface.

## Results

### Characterization of CNT–ND hybrid

**Figure 1:**
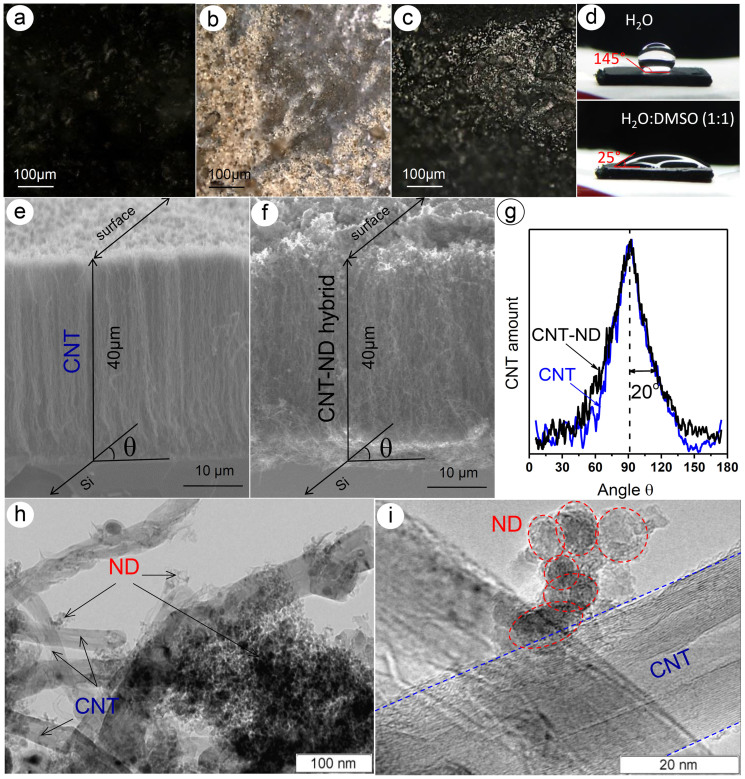
Optical images of surface of CNT array (a), ND powder (b), CNT–ND hybrid (c), and droplet of deionized water (d, top) and DMSO–water (d, bottom) on the surface of CNT array. Side-view SEM images of array of aligned CNTs (e) and CNT–ND hybrid material (f). Angular dependence of CNT orientation in array before and after the ND deposition obtained from the Fourier transform analysis of magnified SEM images (g). TEM (h, i) images of CNT–ND hybrid material.

Optical microscopy images taken from the surface of samples revealed that the top of CNT array is black (Fig. 1a), while the color of ND powder is changed from light-gray to dark-brown (Fig. 1b). The surface of the CNT–ND hybrid reflects the light (Fig. 1c) indicating that NDs were successfully deposited on the array top. Infiltration of water and DMSO–water into aligned CNT array was examined locating a droplet on the array surface (Fig. 1d). Contact angle for the water droplet was equal to 145° and it was unchanged over 15 min. The first droplet of a mixture of DMSO and water taken in equal volume ratio penetrated into the CNT array in less than a second, however we managed to measure the contact angle of the next droplet, which was 25°. These experiments clearly demonstrate improvement of the CNT wettability with addition of DMSO to water.

Analysis of side-view SEM micrographs showed that CNTs in the initial sample are well aligned (Fig. 1e), and immersion of the CNT array in the DMSO–water suspension of NDs did not destroy the vertical orientation of CNTs relative to the Si substrate (Fig. 1f). The thickness of the CNT–ND hybrid is ~40 µm that is close to the thickness of the initial CNT array (Fig. 1e). The NDs are located on the surface of CNT array and in space between the nanotubes. Degree of CNT alignment in the samples was evaluated from the Fourier transform analysis of side-view SEM micrographs obtained at appropriate magnification. Details of the procedure are described in Ref. 30, 31. The CNTs in the hybrid have the angular distribution similar to that in the initial array with an average angle of deviation from the normal to the substrate of ~20°.

The gain in the weight of the sample after NDs deposition was ~40%. Because size of the CNT array was changed marginally, we conclude that the deposition of NDs increases the sample density. TEM images indicate that outer diameter of the CNTs is no more than 50 nm, and the average size of primary ND particles is ~5 nm (Fig. 1 h, i). The ND particles mainly form aggregates with a size of more than 200 nm, which are located between the nanotubes. The smaller aggregates with a size of 30 nm, consisting of a few ND particles, are attached directly to the outer walls of CNTs. However, we should mention that the most part of the CNT surface is free from the NDs. Thus, our product is different from the CVD-produced CNT–ND hybrid materials, where the ND particles almost completely covered the CNT surface^12^,^13^.

**Figure 2:**
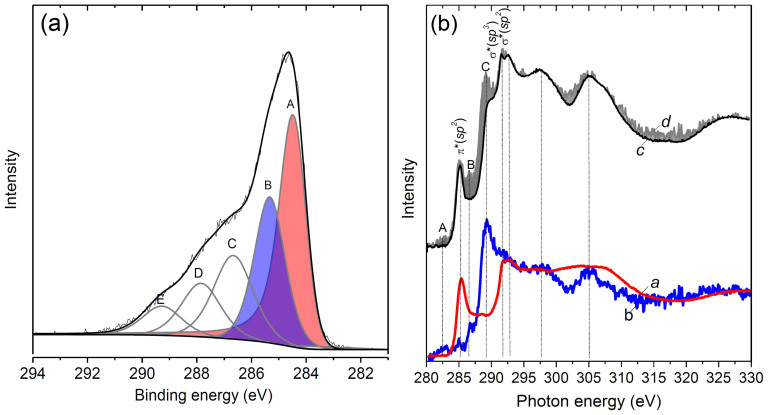
(a) XPS C1s spectrum of CNT–ND hybrid material. The spectrum was fitted with five components using Gaussian/Lorentzian product function with a Doniach-Sunjic high energy tail. (b) C K-edge NEXAFS spectra of CNTs (*a*), NDs (*b*) and CNT–ND hybrid material (*c*). The superposition of the spectra of CNTs and NDs taken in a ratio of 1:1 (*d*).

Overall XPS spectrum of the CNT–ND material showed signals of carbon as a dominant element, and oxygen (14 at%), nitrogen (1 at%), iron (1 at%), and silicon (3 at%) (see Supplementary Fig. S1 online). The XPS C1s spectrum of the hybrid is presented in Fig. 2a. The most intense components A and B located at 284.5 and 285.4 eV are assigned to the *sp*^*2*^–hybridized carbon atoms constituting the CNT walls and the *sp*^*3*^–hybridized carbon atoms from the ND particles. Thus, from the analysis of the XPS data, the hybrid material is a composition of weakly interacting CNT and ND constituents. The high-energy spectral components C, D, and E at 286.7, 287.9 and 289.3 eV likely correspond to the oxygen-containing species existing on the ND surface^32^. The component C is attributed to hydroxyl (C–OH) or ether (C–O–C) groups, while the components D and E are arisen from carbonyl (C = O) and carboxyl (COOH) groups, respectively. According to the fitting of C1s curve, the surface of the CNT–ND hybrid consists of ~40% of graphite-like carbon, ~25% of diamond-like carbon, and ~35% of carbon bonded with oxygen.

To examine the electronic structure of CNTs and NDs in the hybrid, the comparative analysis of the NEXAFS spectra near the C K-edge of CNT–ND sample, CNTs and NDs was performed (Fig. 2b). The CNT spectrum showed two main resonances at 285.4 and 291.7 eV arisen from 1s→π* and 1s→σ* transitions within the *sp*^*2*^–hybridized carbon atoms (curve *a* Fig. 2b). The spectral feature located at 288.6 eV corresponds to the oxygenated carbon atoms. Relatively lower intensity of this feature is indicative of low functionalization of the CNTs. The ND spectrum exhibited the absorption edge at 289.4 eV assigned to the 1s→σ* transitions within the *sp*^*3*^–hybridized carbon and the dip at 302 eV corresponding to a second absolute gap in the diamond band structure (curve *b* in Fig. 2b). Oxygen-containing groups located on the ND surface contribute to the peaks B and C at 286.7 and 289.4 eV^33^. Additionally, the spectrum of NDs shows a weak π* resonance at 285.1 eV indicating the presence of graphite-like species on the ND surface and low-intensity feature A at 282.7 eV, which may be attributed to the *sp*–type amorphous carbon^34^. It should be noted that an unambiguous interpretation of the last feature is still to be done.

NEXAFS C K-edge spectrum of the CNT–ND material exhibited the π*(*sp*^*2*^) and σ*(*sp*^*2*^) resonances characteristic of CNTs, as well as the σ*(*sp*^*3*^) resonance and the gap at 302 eV typical for NDs (curve *c* in Fig. 2b). The surprising result is that the π*(*sp*^*2*^) and σ*(*sp*^*2*^) resonances in the CNT–ND spectrum are narrower and more apparent than those in the spectrum of the initial CNTs. It is likely that the ND deposition process decreases amount of amorphous carbon on the CNT array surface. Relative concentration of the *sp*^*2*^ carbon atoms in the CNT–ND hybrid was estimated from the NEXAFS data using a procedure described in Ref. 35. The portion of the *sp*^*2*^ carbon fraction, %*sp*^*2*^, was obtained dividing the ratio of π*(*sp*^*2*^) and σ*(*sp*^*2*^) peak intensities for the CNT–ND hybrid by that for the CNTs: 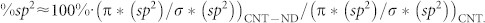


For numerical integration, the energy ranges of 282.5–286.7 eV and 291–295 eV were used to represent contributions of the π* and σ* states to the spectra. The concentration obtained of the *sp*^*2*^ carbon in the CNT–ND hybrid is ~50%. To more clearly elucidate the difference between the electronic structure of the CNT–ND hybrid and its constituents, we constructed a profile summing up the NEXAFS spectra of CNTs and NDs in ratio of 1:1 (curve *d* in Fig. 2b). The resultant profile is close to that of CNT–ND spectrum in the region of π*(*sp*^*2*^) and σ*(*sp*^*2*^) resonances and the post-edge region. The most significant difference (gray-highlighted in Fig. 2b) between the plotted spectrum and the measured spectrum is the absence of the peaks A and B at 282.7 and 286.7 eV and substantially lower intensity of the peak C at 289.4 eV in the latter spectrum. All of these peaks arise from the ND spectrum and correspond to graphite-like species and oxygenated carbon atoms located on the ND surface. Suppression of the peaks provides evidence of the modification of the ND surface, namely, decrease of amount of oxygen-containing groups in the hybrid material. Analysis of the NEXAFS data does not indicate formation of any covalent bonding between constituents of the CNT–ND hybrid, while it shows the change in the chemical state of the surface of deposited ND particles.

Partial defunctionalization of the ND surface in the hybrid was confirmed by FTIR spectroscopy (see Supplementary Fig. S2 online). Analysis of the spectra revealed that purification of the NDs in DMSO–water mixture occurs only in the presence of CNTs. It is difficult to propose the definitive mechanism of interaction between NDs and CNTs, but it is obvious that oxygen-containing groups are involved in this interaction. It can be supposed that a part of chemically active functional groups on the surface of NDs are bound to defect states or hydrocarbon species located on the CNT surface yielding a non-covalent interaction between partially defunctionalized NDs and CNTs. Quantum-chemical modeling reveals moderate electron transfer from a hydrogen-terminated diamond surface to an adsorbed nanotube, while there is no charge transfer between a clean diamond surface and the nanotube^36^. However, the clean diamond surface can form chemically stable interface with an open-ended nanotube^37^.

**Figure 3:**
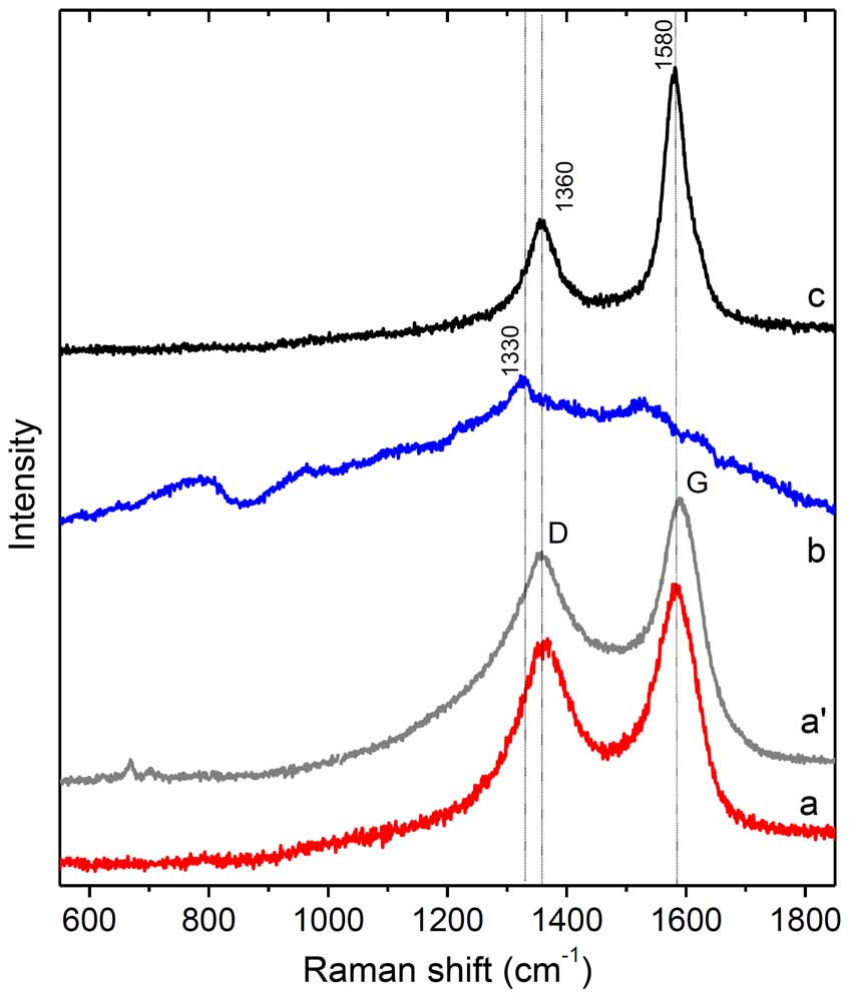
Raman spectra of untreated CNTs (a), CNTs treated by DMSO–water mixture (a'), untreated NDs (b) and CNT–ND hybrid material (c).

Raman spectra of the CNTs, NDs and CNT–ND sample are compared in Fig. 3. The CNT spectrum has wide G and D bands at ~1580 and ~1360 cm^−1^ (Fig. 3a). The G band corresponds to the in-plane tangential stretching of the *sp*^*2*^–hybridized carbon atoms, while the D band is believed to be associated with the disorder-induced double-resonance. A ratio of D band integral intensity (I_D_) to G band integral intensity (I_G_) is often used to evaluate defectness of graphitic materials. Relatively high intensity of the D band in the spectrum of CNTs (I_D_/I_G_ ratio is 1.2) indicates a presence of large amount of non-graphitic carbon in the CNT array, and this is typical for the CNTs produced by CVD technique^38^. The Raman spectrum of NDs shows three prominent features. The weak peak at ~1330 cm^−1^ corresponds to the symmetric vibrations of the *sp*^*3*^–hybridized carbon atoms in diamond lattice, the peak at ~1530 cm^−1^ is a down-shifted graphite G peak, and the wide shoulder at 1600–1800 cm^−1^ originates from oxygen-containing groups located on the ND surface (Fig. 3b). Mochalin et al. had demonstrated that hydroxyl and carboxyl groups may contribute to this shoulder^39^, and presence of such groups on the ND surface was revealed from the analysis of XPS and FTIR spectra (Fig. 2b and Supplementary Fig. S2b online). Low intensity of the diamond peak and presence of the features, corresponding to the graphitic and surface species, denote the low crystallinity and large surface area of ND particles. The Raman spectrum of the CNT–ND sample is dominated by the graphitic G and D bands at ~1580 and ~1360 cm^−1^, while the diamond band at ~1330 cm^−1^ is overlapped by the larger D band (Fig. 3c). The bands in the spectrum of the hybrid are narrower than those in the CNT spectrum, and, moreover, the I_D_/I_G_ ratio decreases to 0.7. Based on these data, we conclude that the amount of defective carbon on the surface of CNT–ND sample is less than that on the CNT array surface. It might be supposed, that small carbon particles located on the outer surface of CNTs were washed due to immersing of the array into solution. However, no similar changes were observed in the Raman spectrum of CNTs treated by DMSO–water mixture (Fig. 3a). So, we conclude that ND particles somehow participate in the process of purification of the CNT array surface from small carbon impurities. It is possible, that DMSO helps penetration of ND agglomerates into intertube space and the agglomerates mechanically move small carbon particles from the surface to the array depth. Deposition of ND particles yields 40% increase in density of the array that confirms good penetrating capacity of the used ND suspension.

### Field emission and luminescence of CNT–ND hybrid

**Figure 4:**
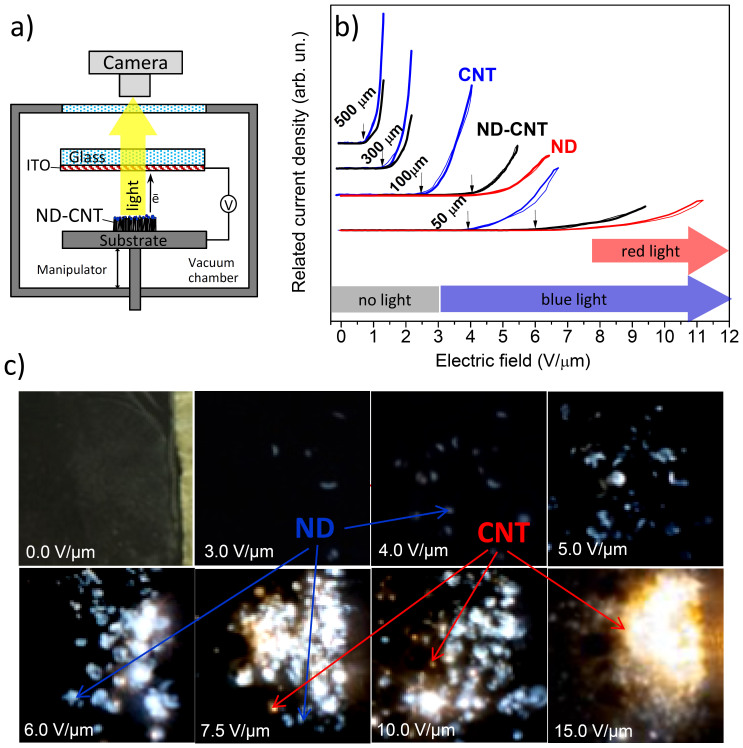
Scheme of set-up for field emission and electroluminescence measurements (a). *I-V* curves for CNTs (blue), NDs (red) and the CNT–ND hybrid material (black) measured at 50, 100, 300 and 500 µm (b). Arrows indicate the threshold field. Images of light emission from surface of the CNT–ND hybrid material at electric field from 0 to 15 V/µm (c).

Since anisotropic materials based on the aligned CNTs and NDs have potential application as cold cathode in light-emitting devices, a study of field electron emission and electroluminescence of the hybrid prepared were performed. A scheme of the measurements is shown in Fig. 4a. The current *vs* voltage (*I–V)* curves of field emission from the initial CNTs, NDs, and obtained CNT–ND hybrid measured at various distances (50, 100, 300 and 500 µm) from the anode surface are compared in Fig. 4b. The applied field is given as the macroscopic electric field defined by the ratio of the voltage to the given interelectrode distance. The value of voltage threshold was determined at the emission current density of 10^−8^ A/mm^2^. The current from the CNT sample appear at 0.8, 1.4, 2.2 and 3.9 V/µm at the distance of 500, 300, 100 and 50 µm, respectively. Such low electric field threshold is characteristic of the arrays of aligned multiwall CNTs^40^,^41^,^42^. Increase in the threshold value with a decrease of the interelectrode distance is attributed to the change in the enhancement factor due to screening effects^37^. Low electrical conductivity and small aspect ratio of ND particles cause reduction of the threshold up to ~4.4 V/µm at the interelectrode distance of 100 µm and ~6.5 V/µm at the 50–µm–distance. For distance of 100 µm the maximum current density for the NDs was 2.5 A/mm^2^. That is three times lower, than current density of 7.3 A/mm^2^ for the CNT sample. It is believed that in the case of NDs, electron emission originates from their modified surface (graphite-like species and functional groups)^43^,^44^. The threshold field for the CNT–ND hybrid is ~0.9 V/µm at 500 µm and ~1.6 V/µm at 300 µm that is very close to that for the initial CNT array. Current density for the hybrid is twice lower than that for the initial CNTs. We believe that a part of CNTs does not emit due to their covering by the ND particles. However, at the closest interelectrode distances of 100 and 50 µm, the voltage thresholds of 4.1 and 6.0 V/µm are slightly lower, than that for the ND powder. The behavior of *I–V* curves for the hybrid material indicates, that at low electric field, electron emitting centers are mainly CNTs, while at electric field higher than 3.5 V/µm, electron emitting located on the top surface of CNT array are mainly ND particles and the thickness of deposited ND layer is large enough to screen the electron emission from the CNTs.

Registration of the electroluminescence glow was done through a transparent anode. Optical image of the surface of a CNT–ND cathode located in measurement camera is presented in upper-left corner of Fig. 4c. Remaining images were obtained when an electric field was applied to the cathode. It should be noted that light emission was not observed until the field was less than ~3.0 V/µm. At this field value, our optical system was able to register blue spots on the CNT–ND surface. Based on the *I–V* data presented in Fig. 4b, we assume that the NDs are responsible for the luminescence. The number and brightness of luminous blue spots increase with the applied field. Red bright dots appeared at 7.5 V/µm should be attributed to the CNT burning.

**Figure 5:**
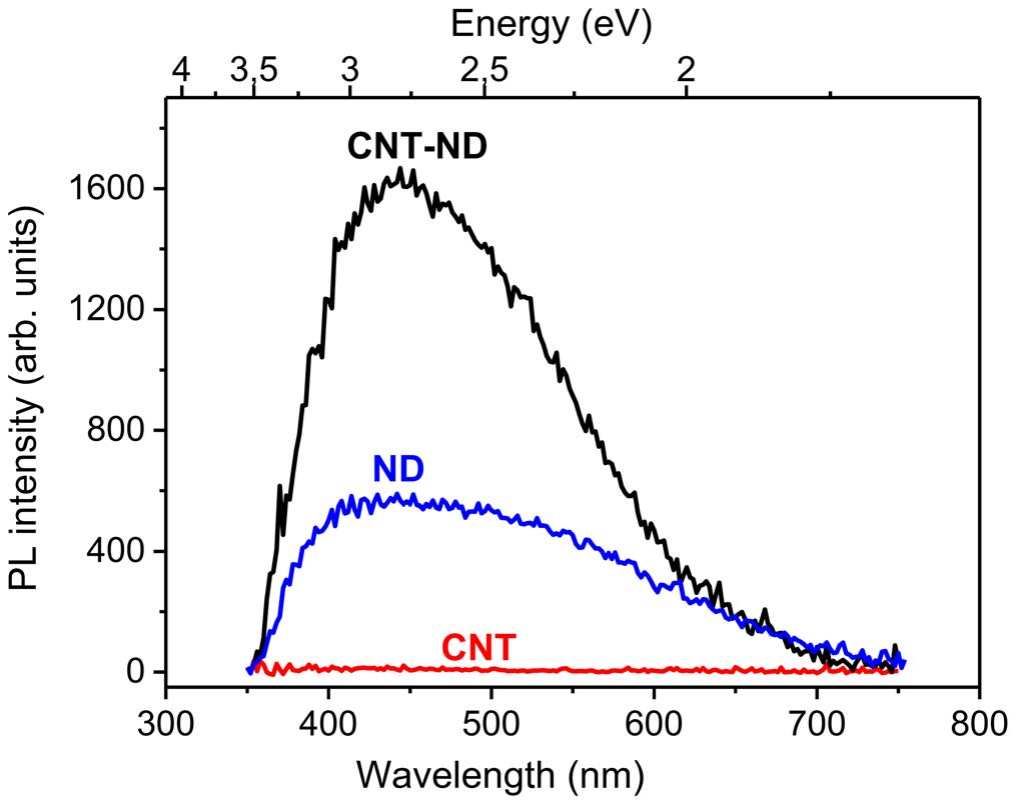
Photoluminescence spectra of CNT–ND hybrid material, CNTs and NDs treated by a DMSO–water mixture.

To understand the origin of the emitting centers, room-temperature photoluminescence spectra of CNT–ND hybrid material, CNT array treated by a DMSO–water mixture, and NDs deposed on a Si substrate from a DMSO–water suspension were measured (Fig. 5). The spectrum of CNTs shows no peaks in the spectral range of 300–800 nm, because multiwall CNTs are metallic. The spectra of CNT–ND hybrid and NDs have a broad peak from 380 to 700 nm with a full width at half maximum of ~200 nm for both spectra. The spectrum of NDs shows the blue-green band with near constant intensity in a region from 400 to 600 nm. Blue-green photoluminescence and electroluminescence have been previously observed for the diamond films^45^,^46^. It is believed that blue luminescence arises due to nitrogen contaminations^45^, while C = O surface groups are responsible for green emission^28^. The spectrum of the CNT–ND hybrid has intense blue band centered at 440 nm (2.8 eV) with a tail in the green emission region. Decrease in the green emission from the hybrid material as compared to the initial NDs is likely caused by partial removal of carbonyl groups from the ND surface as the result of the hybrid synthesis. Intensity of the photoluminescence of the CNT–ND hybrid is roughly twice higher than that of the NDs. Thus, we believe that blue glow observed on the CNT−ND hybrid surface under applied electric field is due to electroluminescence of the ND particles located at the CNT tips.

The NDs deposited on a copper substrate produced no glow at an electric field as high as 15 V/µm (See Supplementary Fig. S3a online). Kim at al reported that field emission electroluminescence from polycrystalline diamond films is observed only at the field higher than 50 V/µm^29^. Due to high aspect ratio of a CNT, electric field is strongly enhanced at its tip, and high field strength helps in excitation of the valence electrons to unoccupied levels within a ND particle. The back relaxation of electrons causes photon emission. High intensity and stability of luminescence observed for the CNT–ND material as compared to the NDs can be explained by injection of electrons from a conductive CNT into semiconductive NDs attached to the tip of the CNT. The nature of red bright spots in the electroluminescence images of CNT−ND hybrid (Fig. 4c) could be attributed to radiation from resistively heated multiwall CNTs. Actually, the same glow was observed for the surface CNT array treated by DMSO–water mixture (without NDs) at the field of 8 V/µm (see Supplementary Fig. S3b online). Previously, light emission from CNTs induced by the field emission current was explained by the Joule heating of CNTs up to temperatures of 1500–2000 K^47^. As the result of this process, CNTs can be destroyed^42^. XPS examination of the CNT–ND hybrid material after the field emission measurements showed the same set of the peaks (Supplementary Fig. S4 online) as the spectrum of the hybrid before the experiments (Fig. 2a). One of the main differences in the spectra is a higher ratio of *sp*^*3*^- to *sp*^*2*^-carbon in the hybrid after application of the electric field. It is more likely that spectrum of the sample after application of field emission was measured at another spot, than that of the sample before experiment. We suggest that high amount of *sp*^*3*^- diamond like carbon was observed in the spectrum of sample after measurements just because ND particles non-uniformly cover the CNT array surface. Moreover, the peak E at 289.3 eV has too low intensity due to partial decarboxylization of NDs during the field emission. This could explain suppression of green emission observed in the photoluminescence spectrum of the CNT–ND hybrid.

## Discussions

Anisotropic CNT–ND hybrid material was successfully synthesized treating the array of vertically aligned CNTs by a suspension of NDs in a DMSO–water mixture. The developed method allows varying the ratio of constituents, which can be as large as 1:1. Analysis of the CNT–ND hybrid using microscopy methods shows that ND agglomerates are located on the array surface and partially fill the space between CNTs. Penetration of ND particles into the depths of the CNT array is realized owing to wetting property of DMSO. It was found that NDs take a part in the purification of the CNTs array surface from carbon impurities, formed during the CVD synthesis. Furthermore, DMSO is likely to provide interaction between functional groups located on the ND surface and CNTs that result in partial defunctionalization of NDs. XPS and NEXAFS methods reveal that CNTs and NDs preserve their identity in the hybrid. CNT–ND hybrid material possess field emission behavior of CNT array at an electric field less than 3 V/µm, while under higher electric field, the ND particles located on the CNT tips emit electrons. Despite of non-covalent interactions between the hybrid constituents, the ND particles located on the CNT tips emit photons, which are blue and green colored. Removal of oxygen-containing groups from the ND surface as the result of the hybrid synthesis and field emission experiments causes a quenching of green light emission. We believe that the anisotropic CNT–ND hybrid materials will be attractive for development of bright blue-light sources.

## Methods

### Materials

CNT arrays were grown on *p*-doped Si(100) substrates of size 10 × 10 mm^2^ using an aerosol-assisted catalytic CVD method. The details of the method are described elsewhere^48^. Reaction mixture obtained from a solution of 2 wt% ferrocene in toluene was dispersed into a volume of tubular reactor via injector. The pyrolysis was performed at ~800°C and atmospheric pressure in an Ar flow (200 cm^3^/min) during an hour. A ND-containing soot was produced by detonation of trinitrotoluene and hexogen in the absence of oxygen. ND particles were extracted from the detonation soot using NaOH accordance to with the technology developed by the “Real-Dzerzhinsk” Limited Liability Company (Russia). The purified NDs were fractionated via centrifugation of ND suspension in an aqueous solution of NaCl following procedure described in Ref. 49. The fraction of NDs sized ranging 4–200 nm was taken to prepare a hydrosol by dissolving the powder (5 wt%) in deionized water. The final ND suspension was obtained by diluting the hydrosol by DMSO in a ratio of 1:1. The CNT–ND hybrid was prepared by immersing the CNT array into the ND suspension for 5 min. After that the sample was dried at ambient conditions for 48 hours.

### Characterization

Morphology of the samples was investigated by optical microscopy on an Olympus BX51 microscope, scanning electron microscopy (SEM) on a JEOL JSM–6700F microscope, and transmission electron microscopy (TEM) on a JEOL 2010 microscope. To prevent disintegration of the hybrid material during a sonication procedure commonly used for the TEM specimen preparation, a portion of sample was scratched from the Si substrate using a sharp tungsten tip, and the collected material was deposited on a copper TEM grid. XPS measurement was performed using a Specslab PHOIBOS 150 spectrometer with a monochromatic Al K_α_ (hν = 1486.6 eV) radiation. The C1s spectrum was measured with high resolution at the constant pass energy of an electron energy analyzer of 20 eV and the energy step of 0.1 eV. The curve fitting was carried out using a Gaussian/Lorentzian product function with a Doniach-Sunjic high energy tail (asymmetry factor α is not zero only for the component at 284.5 eV) with CasaXPS 2.3.15 software. The C K-edge NEXAFS spectra were recorded in the total-electron yield mode at the Berliner Elektronenspeicher ring für Synchrotronstrahlung (BESSY II) using radiation from the Russian-German beamline and with the MUSTANG experimental station. The monochromatization of the incident radiation was ~80 meV. The spectra were normalized to the primary photon current from a gold-covered grid recorded simultaneously. The FTIR spectra were acquired using an IFS–85 «Bruker» spectrometer in a range from 400 to 4000 cm^−1^. A sample (1 mg) was mixed with KBr powder (100 mg) and then the mixture was pressed into a pellet. The Raman analyses were performed using a Spex 1877 triple spectrometer with a 488 nm Ar^+^ laser.

### Filed emission, PL and EL measurements

Field electron emission and electroluminescence measurements were carried out in a vacuum chamber at ~5·10^−4^ Pa at room temperature using a home-made set-up^50^. Si substrate with a sample was attached to a negative electrode by carbon conductive tape, while ITO (indium tin oxide) coated glass was used an anode. The distance between the surface of a sample and the anode was changed from 50 to 500 µm. The current–voltage (*I*–*V*) dependence was obtained by applying a dc voltage of up to 1000 V. A sawtooth voltage regulated the electric field with a frequency of 0.1 Hz. For the electroluminescence glow images were measure using a square wave with amplitude 1.5 kV and frequency 200 Hz. Photographs of the surface of CNT–ND cathode were taken through a transparent window in the measuring cell using a photo camera with an exposure time of 10 sec. The steady photoluminescence of the sample was excited by a continuous He–Cd laser with a wave length of 325 nm and a power density of 0.5 W/cm^2^. The measurements were performed at room temperature.

## Author Contributions

Yu.V.F., L.G.B. and A.V.O. promoted the idea of the manuscript, wrote the main manuscript text, and prepared all figures, M.A.K. measured the electroluminescence and field emission characteristics, D.V.G. synthesized the carbon nanotube arrays, I.P.A. performed XPS measurements, D.V.V. provide measurements of the NEXAFS spectra, A.P.P. and V.S.B. developed and realized the method to synthesize the hybrid sample, D.V.G. performed photoluminescence spectra. All authors discussed the results and reviewed the manuscript.

## Supplementary Material

Supplementary Information Supplementary Information
