# Supplementary material for: Field emission luminescence of nanodiamonds deposited on the aligned carbon nanotube array
Source: Sci Rep. 2015 Mar 23;5:9379. doi: 10.1038/srep09379 (PMC4369750; doi:10.1038/srep09379)
Supplement: Supplementary Information [file srep09379-s1.pdf]

Supplementary Information for:

Field emission luminescence of nanodiamonds deposited on the aligned carbon nanotube array

Yu.V. Fedoseeva[1,2,], L.G. Bulusheva[1,2], A.V. Okotrub[1,2], M.A. Kanygin[1], D.V. Gorodetskiy[1], I.P. Asanov[1,2], D.V. Vyalikh[3], A.P. Puzyr'[4], V.S. Bondar'[4]*

[1]Nikolaev Institute of Inorganic Chemistry SB RAS, Novosibirsk 630090, Russia

[2]Novosibirsk State University, Novosibirsk 630090, Russia

[3]Institute of Solid State Physics, Dresden University of Technology, D-01062 Dresden, Germany

[4]Institute of Biophysics SB RAS, Krasnoyarsk 660036, Russia

E-mail: fedoseeva@niic.nsc.ru (Dr. Yuliya V. Fedoseeva)

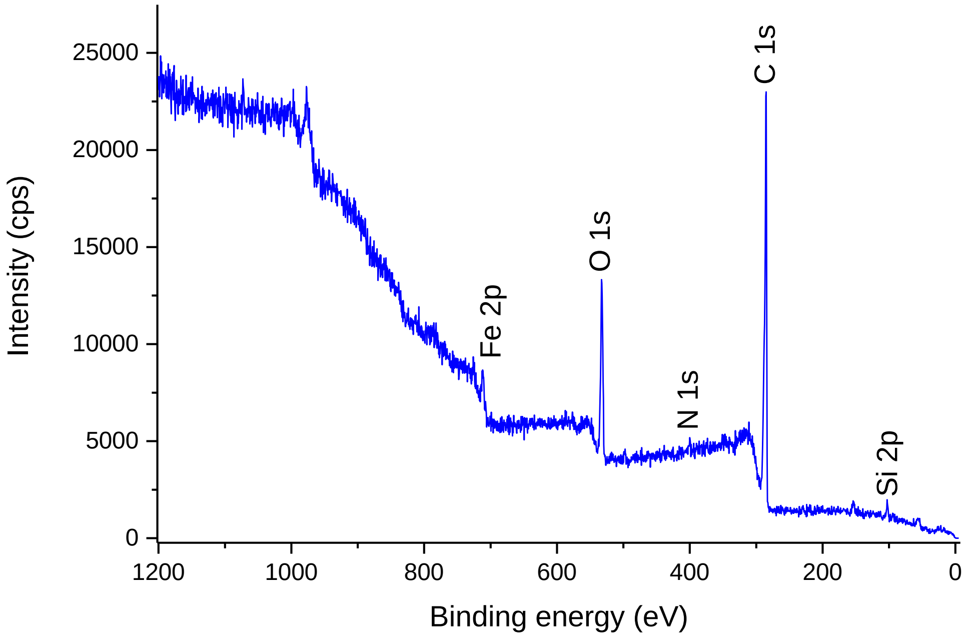

Figure S1: XPS overall spectrum of CNT–ND hybrid material.

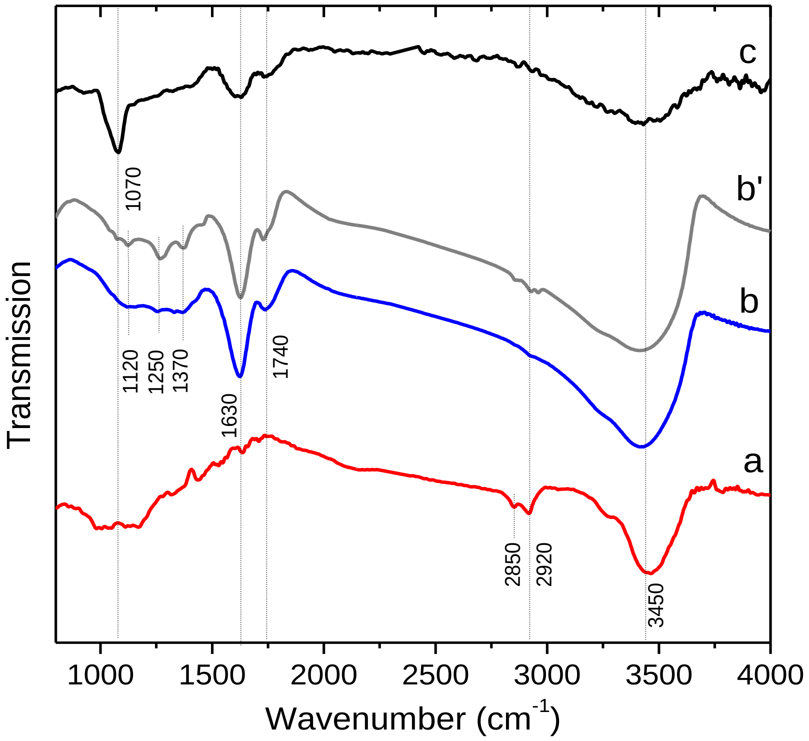

Figure S2: FTIR spectra of CNTs (a) and NDs (b) treated by DMSO–water mixture, untreated NDs (b') and CNT–ND hybrid material (c).

The FTIR spectrum of CNTs treated by a mixture of water and DMSO, taken in equal proportions, consists of a strong and broad absorption band at 3450 cm^{-1} attributed to the vibration of hydroxyl groups or/and adsorbed water molecules. (curve *a* in Fig. S2). The bands between 1000 and 1700 cm^{-1} are ascribed to vibrations of carbon cage and C–O–C groups, as well as deformation vibrations of CH_x groups. The C–H stretching vibrations in CH_x groups are at 2850 and 2920 cm^{-1}. The well-resolved bands at 1630 and 1740 cm^{-1} in the FTIR spectrum of NDs correspond to stretching C–O vibrations in carbonyl, carboxyl, and ketone groups, and bending O–H vibrations (curve *b* in Fig. S2). The bands in the region of 1000–1300 cm^{-1} is assigned to C–O–C vibrations in ether, phenolic, and epoxy groups. The FTIR spectrum of the NDs treated by a DMSO–water mixture almost completely reproduces the spectrum of untreated NDs (curve *b'* in Fig. S2). The FTIR spectrum of CNT–ND hybrid material (curve *c* in Fig. S2) exhibits the bands corresponding to the C–O–C, C–O and O–H vibrations. Intensities of these bands are lower than those observed in the reference spectra of NDs and CNTs. The higher intensity has the band located at 1070 cm^{-1}, which may be assigned to the stretching vibrations in

ether groups or C–O stretching vibrations in primary alcohols and/or to the S=O stretching vibrations in the residual DMSO molecules. Note, that XPS reveals no presence of sulfur in the hybrid.

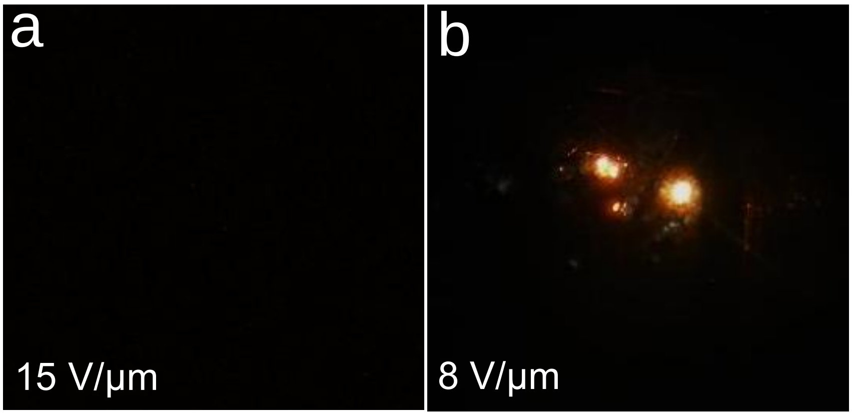

Figure S3: Images of the surface of NDs deposited onto a Cu substrate at an electric field of 15 V/µm (a) and CNT array treated by DMSO−water mixture at an electric field of 8 V/µm (b).

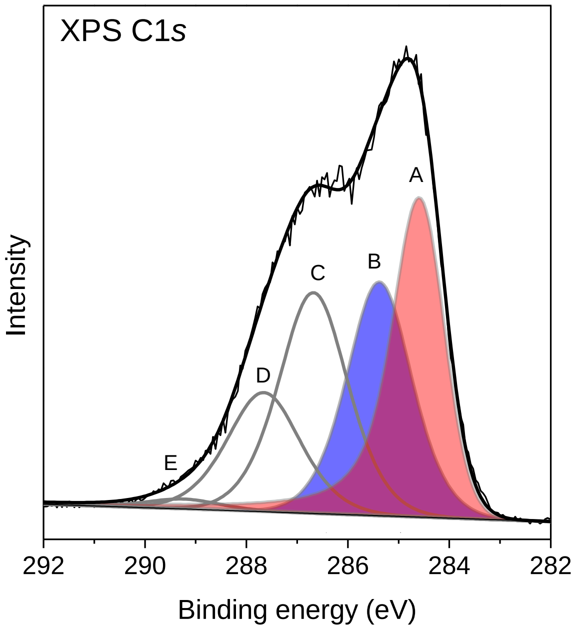

Figure S4: XPS C 1s- spectrum of the CNT–ND hybrid material after field emission measurements.
